# Dental Caries Status and its Related Factors in Iran: A Meta-Analysis

**DOI:** 10.30476/DENTJODS.2020.82596.1024

**Published:** 2020-09

**Authors:** Mohammad Reza Soltani, Mahsa Sayadizadeh, Sajad Raeisi Estabragh, Kiana Ghannadan, Mahsa Malek-Mohammadi

**Affiliations:** 1 Dept. of Operative Dentistry, School of Dentistry, Ilam University of Medical of Sciences, Iran; 2 Dept. of Pediatric Dentistry, School of Dentistry, Kerman University of Medical Sciences, Kerman, Iran; 3 Dept. of Prosthodontics, School of Dentistry, Rafsanjan University of Medical Sciences, Rafsanjan, Iran; 4 Dept. of Operative Dentistry, School of Dentistry, Qazvin University of Medical of Sciences, Qazvin, Iran; 5 Student Research Committee, School of Dentistry, Shahid Beheshti University of Medical Sciences, Tehran, Iran

**Keywords:** Dental Caries, Dental Decays, DMFT, Meta-Analysis, Iran

## Abstract

**Statement of the Problem:**

Dental caries is a global health issue, which imposes a great deal on individuals and the community.

**Purpose:**

The main purpose of this study was to identify the dental caries status and/or DMFT/dmft data and its related factors in Iran.

**Materials and Method:**

The search performed in the online databases to identify all literature published up to Oct 2018. The random effects model was applied to pool analysis and verses. Funnel plots and Egger test used to examine publication bias. All analyses were carried out with R software version 3.2.1 and STATA (version 11.1).

**Results:**

69 studies selected as eligible for final analysis in which all subjects were in age range less than 18 years old except 4; so, all results and analyzes were only calculated in this age group. The total rate of dental caries was 72.8% (95%CI, 69.2-76.4%) and the mean of dental caries was 2.33 (95% CI, 2.12–2.54) based on DMFT values and 3.86 (95% CI, 3.49–4.22) based on the dmft values. These rates were almost similar in both sexes. In addition, these trend were higher in rural than urban regions. Subgroup analysis found a direct relationship between DMFT index and age, while the reverse was true for relationship between dmft and age. The mean dental caries were higher in children with low socioeconomic status, low family income, low educated and unemployed parents, crowded families, excessive carbohydrate intake, and less toothbrushes frequency.

**Conclusion:**

The present study showed high prevalence and experience of dental caries among children and adolescents (<18 years of age) in Iran. This illustrates ineffective oral health national preventive programs and lack of educational measures. New preventive procedures, practical educational programs, and modern therapeutic methods are needed to improve oral health status.

## Introduction

Dental caries, also known as tooth decay, is a dynamic biological process of irreversible destruction of susceptible dental hard tissues because of acids produced by bacterial glycolysis of dietary carbohydrates [ 1 ]. According to the World Health Organization (WHO) report, approximately 60% to 90% of school-aged children and nearly all adults have dental caries at some point in their life span [ 2 ]. Several etiological factors include fermentable carbohydrates, qualitative bacterial components, oral colonization by cariogenic bacteria, a susceptible tooth (host), amount and components of saliva, poor oral hygiene, and time are contributed in the onset and progression of dental caries [ 3 , 4 ]. Usually, dental caries in epidemiologic surveys evaluated according to the WHO criteria using dmft (the decayed, missing, filled teeth for primary teeth) and DMFT (the decayed, missing, filled teeth for permanent teeth) indices, which measure caries at cavitation level [ 5 , 6 ]. Dental caries can cause pain, inflammation and gingival bleeding, abscess formation, tooth loss, and subsequently loss of available space in the arch [ 7 ]. It is also expensive to treat and may cause oral malodor, leads to lost productivity, causes harm to nutrition and affects overall health and quality of life [ 8 ]. Furthermore, it is linked to some systemic diseases such as cardiac problems, stroke, and respiratory disease since it may cause chronic infections in the body [ 9 ]. Several studies have shown that dental caries affects all ages, races, and socioeconomic groups and reported the correlation between children's dental caries with some demographic and socioeconomic factors including age, sex, level of parents’ education, the family income, and socioeconomic backgrounds [ 10 , 11 ]. Despite the application of multiple health programs to improve oral health, dental caries is still considered as a global health concern; therefore, it is necessary to assess the strategies for primary prevention of this disease and to implement health promotion programs. The measurement of oral health status and conducting accurate and up-to-date research is important for understanding natural history and biological processes of a disease for projecting and assessing health services. There are a number of studies with various populations done in Iran regarding the prevalence of dental caries or DMFT/dmft data; therefore, an overall estimation of the prevalence is needed to help understanding the status of caries by synthesizing available studies in our country more clearly. In addition, the format of a systematic review is very effective in collecting a large amount of data, understanding the breadth and quality of conducted studies, and analyzing simultaneously. With this background, performing a systematic review and meta-analysis seems to be necessary. To the best of our knowledge from indexed literature, a meta-analysis of dental caries prevalence, experience, or incidence in Iran has yet to be reported. Therefore, the main purpose of this study was to identify the dental caries status and/or DMFT/ dmft data in Iran. We also considered the effect of demographic and socioeconomic factors on oral health.

## Materials and Method

### Search methods for identification of studies

Electronic searches were carried out for related Persian and English articles (up to October 2018) with existent documents in national and international online databases. Searching was done using keywords and search terms including «dental caries», «dental decays », «dental missing», «dental filling», «DMFT», «dmft», «dft», «dmfs», «dfs», «Iran», «Iranians» and «Persia» through international databases: PubMed, the Scopus databases, ScienceDirect, and Google Scholar. The national databases (SID, Noormag, Magiran, Iranmedex, and Irandoc) were also searched using all probable combinations of the Persian equivalents of identified keywords. We completed our electronic search with hand searches of reference lists of all primary studies and review articles to identify any studies that could have remained unidentified in the previous step. Only published and accessible papers were considered.

### Inclusion and exclusion criteria

Records of all references obtained through the search strategy were combined in the reference management software, EndNote X4 (Thomas Reuters, Philadelphia, PA, USA), and duplicate items were deleted using the features of this software. All types of studies, including observational longitudinal, cross-sectional, cohort and case-control studies reporting dental caries prevalence, experience, or incidence in Iran were reviewed. The inclusion criteria were: original studies, studies including caries prevalence and/or DMFT/dmft data; studies conducting in healthy participants who had not previous history of systemic disease and were not being under orthodontic treatment; studies measuring untreated caries and/or DMFT/dmft data through clinical examination by appropriately qualified practitioner/researchers or through health records databases were included. Only studies fulfilling all of these criteria and published in full text were included in the qualitative and quantitative synthesis. In cases of multiple publications from the same population or cohort, only the largest study was included. Studies that were meta-analyses or systematic considerations, and those that presented insufficient data were excluded.

### Data Extraction

The data and information were extracted from eligible articles based on a standard protocol. Study characteristics (the name of first author, publication year, the year and place of the study), demographic features (sample size, age group); the dental variables, socioeconomic parameter, and study type were extracted from each trial. Reported estimates for dental caries were the prevalence/incidence of untreated caries (DMFT/dmft being >0), the prevalence of caries experience (percentage of a population with any caries experience), the caries experience (average DMFT/dmft indexes). Literature review identified key confounders that affect the caries incidence; these items included age, sex, socio-economic status, parent’s educational level, parent’s occupation, the number of children in family, dental visits, and so on. To explore the effects of these factors on the caries incidence, we extracted the reported DMFT/dmft values between the lowest and the highest reported confounder factors and assessed the differences between them. Next, data extraction forms were designed, filled out, and imported into Microsoft Excel. The articles’ authors were contacted for supplementary data or further elucidation, if data were missing or for clarifications.

### Quality assessment of the selected studies

The quality assessment of the included studies was evaluated using the Newcastle–Ottawa Scale (NOS) checklist; NOS assess the domains of selection bias, comparability of groups and attrition bias, and ascertainment of exposure and outcomes [ 12 ]. Study quality was graded on a scoring system. The NOS ranges from zero to nine stars; studies with NOS scores of less than 3, from 4 to 6, and more than 7 were considered as having low, moderate, and high methodological quality, respectively. Two reviewers independently performed search process, selection of studies, data extraction, and quality assessment and their findings and results were compared later. Disagreements were resolved by group discussion.

### Data Synthesis and Analysis

One of the main objectives of this study was to evaluate the prevalence/incidence of caries; therefore, the overall prevalence of dental caries and reported DMFT/dmft values in different studies were extracted to enable quantitative synthesis and analysis. The binomial distribution used to calculate the variance in each study and the weighted mean was used for a combination of prevalence rate in different studies. Each study was given a weight equal to its inverse variance.

To explore the effects of confounder factors on the caries incidence, only studies reporting of DMFT/dmft mean were used for quantitative data synthesis. The rates and mean values with a confidence interval of 95% were computed as the effect measure for both individual trials and pooled estimates. Statistical heterogeneity was evaluated in studies using chi-squared test and I^2^ index. In this meta-analysis, due to the significant heterogeneity of the studies, the random effects model was applied to pool analysis and verses. We undertook subgroup analyses based on sex, age, and region to explore the reasons for heterogeneity. Integrated estimations and the related confidence interval of 95%were evaluated using forest plots as visuals. Funnel plots and Egger test were used to check the possibility of publication bias. Sensitivity analyses were performed to control for the effects of imputing data and to assess the effects of possible publication or reporting bias. Significance was set at *p*< 0.05 as valid for heterogeneity tests. All analyses were carried out with comprehensive meta-analysis R software version 3.2.1 and STATA (version 11.1).

## Results

### Selected Articles

**Figure 1:**
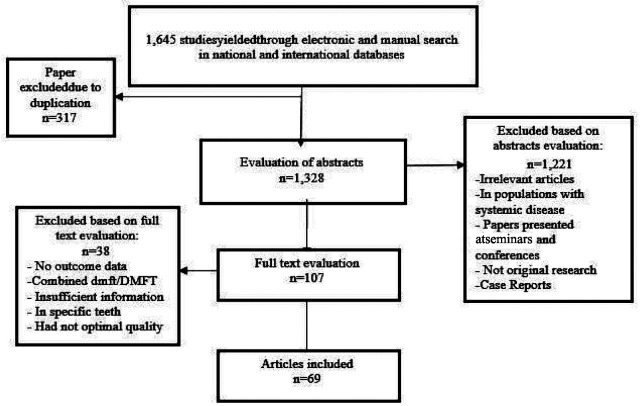
Flow diagram of dental caries status and its related factors in Iran.

A flowchart describing the systematic review search results is presented in Figure 1. The literature searches yielded 1,645 articles, of which 317 papers were repetitive and removed from further consideration. We also excluded 1,221 citations after screening the titles and abstracts as clearly irrelevant to study objectives and for failing to meet the eligibility criteria, leaving 107 papers for full-text review. Of the remainder, another 38 articles after full-text screening were excluded; finally, sixty-nine papers were potentially relevant and eligible for the final analysis (Figure 1).

### Description of the Studies

The studies used in this meta-analysis were published between1998-2018 and all were cross-sectional in design (13,81). The general characteristics and findings of the studies that fulfilled the eligibility criteria are summarized in Table 1. In most reviewed studies, the sampling method was random cluster sampling; information and data were collected through interviews and clinical examination by appropriately qualified practitioner/researchers or through health records databases. Data on the dental caries prevalence, experience, or incidence were available for 54 studies and eight studies presented data related to significant caries (Sic). Fifteen studies analyzed decayed, missing or filled teeth in primary teeth (dmft), thirty-five studies analyzed decayed, missing or filled teeth in permanent teeth (DMFT), and sixteen studies investigated both. The quality assessment of the individual studies is summarized in Table 1.

**Table 1:** Characteristics and information of studies evaluated in this meta-analysis.

Authors (Reference)	Publication year	City	Sample size	Dependent Variable (mean±SD)		NOS
Bashirian [13]	2018	Hamedan	T:988	DMFT:	Dmft:	9
			B:503	T: 0.79±1.53	T: 3.61±3.58	
			G:485	B: 0.63±1.17	B: 4.04±3.78	
				G: 0.96±1.49	G: 3.16±3.31	
				DT: 0.33±0.85	dt: 2.68±3.05	
				MT: 0.009±0.13	mt: 0.60±1.12	
				FT: 0.44±1.04	ft : 0.32±0.93	
Shaghaghian [14]	2018	Shiraz	T:396	Dmft	Dental caries: 69.9% CF: 30.1%	8
			B:202	T: 3.88±3.9		
			G:194	B: 3.95±4.05		
				G: 3.82±3.75		
Esmaeilzadeh [15]	2017	Jolfa, East Azerbaijan	T:146	DMFT:	Dental caries: 92.5%	4
			B:80	T: 4.3±2.93		
			G:46	B: 4.38±2.26		
				G: 4.21±3.60		
				D: 5.37±3.55		
Usefi [16]	2017	Bovair Ahmad	T:460	DMFT:	Dmft:	8
			B:244	T: 0.86±1.31	T: 3.57±3.12	
			G:216	B: 0.76±1.19	B: 3.69±3.25	
				G: 1.0±1.42	G: 3.43±2.98	
				Dental caries: 89.8%		
Jafari [17]	2017	Hashtrood, Tabriz	335	DMFT: 2.09±2.2	Dmft: 4.08±3.51	7
				DT: 3.34±1.54	dt: 4.77±2.42	
				MT: 1.67±1.19	mt: 1.95±1.28	
				FT: 1.46±0.66	ft : 2.0±1.27	
Mehralian [18]	2017	Qazvin	T:373	DMFT: 3.53±4.22	Dmft: 5.66±4.63	6
			B:206	DT: 3.1±3.57	dt: 4.98±4.32	
			G:167	MT: 0.12±0.43	mt: 0.11±0.38	
				FT: 0.3±0.81	ft : 0.13±0.57	
Amiri [19]	2017	Ahvaz	T:359	dmft:	Dental caries: 87.7%	4
			B:161	T: 2.62±2.15		
			G:198	B: 2.87±2.33		
				G: 2.41±1.97		
				CF: 12.3%		
Sistani [20]	2017	Babol	T:2080	dmft:	G: 4.05±4.02	4
			B:1111	T: 4.01±3.89	CF:26.3%	
			G:969	B: 3.98±3.79	Dental caries: 73.7%	
KhaniVarzegani [21]	2017	Tabriz	T:756	dmft	CF;	7
			B:389	T: 4 (2-8)	T:15.2%	
			G:367	B: 4 (2-9)	M: 17.2%	
				G: 5 (2-8)	F: 13.1%	
Khodadadi [22]	2016	Babol	T:384	dmft:	dt=6.5±4.35	5
			B:204	T: 8.2±7.65	mt= 0.4±1.1	
			G:180	B: 8.5±7.79	ft=1.2±2.2	
				G: 7.6±7.4		
Mahmoudi [23]	2016	Fasa	T:4405 B:2031 G:2347	DMFT: T: 1.26±1.64 B: 1.23±1.74 G: 1.29±1.55 DT: 1.0±1.46 MT: 0.12±0.48 FT: 0.15±0.63	Dmft: T: 4.45±3.43 B: 4.51±3.41 G: 4.40±3.44 dt: 3.32±3.00 mt: 0.97±1.43 ft : 0.16±0.70 SiC: 10.18±2.23 CF: 6.8%	8
Marasouli [24]	2016	Urmia	T:93/ B:70/G:23	DMFT: 2.55	Dmft: 2.5	3
Eskandarizadeh [25]	2016	Kerman	T:300 B:150 G:150	Dmft T: 4.63±2.41 B: 5.07±2.38	G: 4.2±2.4 SiC: 7.34±1.34	6
Golkari [26]	2016	Shiraz	T:335 B:158 G:177	DMFT: 1.22±1.5 G:1.00 B: 1.3 Dmft: 2.8±2.5	Dental caries: 75.5% in primary dentition and 47.5% in permanent dentition	7
Rahimian Emam [27]	2015	Zahedan	100	DMFT: 2.29±1.67		5
Asdagh [28]	2015	Ardabil	T:847 B:403 G:444	DMFT: 1.6±0.1 dmft: 2.74±0.09 DMFS: 3.5±0.1	Dental caries: 79.7% (88.3% in primary dentition and 71.1% in permanent dentition)	7
Alimorad [29]	2015	Bandar Abbas	T:768 B:384 G:384	DMFT: T:1.8±1.87 B: 1.98±1.84 G: 1.61±1.89 DT: 1.74±1.83	MT: 0±0.05 FT: 0.32±1.80 Dental caries: T:65.2% B: 71.4%, G:59.1% (permanent dentition), CF:34.8%	7
	2015	Bandar Abbas	T:768 B:384 G:384	DMFT: T:1.8±1.87 B: 1.98±1.84 G: 1.61±1.89 DT: 1.74±1.83	MT: 0±0.05 FT: 0.32±1.80 Dental caries: T:65.2% B: 71.4% G:59.1% (permanent dentition) CF:34.8%	7
Banihashemi-Rad [30]	2015	Mashhad	T:552 B:161 G:391	DMFT: B:1.25±1.22 G: 1.3±1.3 dmft: B:3.43±2.43 G: 3.28±2.56 CF: 4.1%	Dental caries: 95.5% (85% in primary dentition and 60% in permanent dentition)	7
Bahrolooloomi [31]	2014	Yazd	T:400/ B:200 G:200	DMFT/dmft: T: 5.09±1.95	B: 5.12±2.02 G: 5.07±1.89	6
Sajadi [32]	2014	Sirjan, Kerman	B:700	DMFT: 3.56±2.34	SiC: 6.04±1.32 CF:20.3%	8
Khosravani [33]	2014	Shiraz	974	DMFT: T: 0.94±1.46 B: 0.93±1.49	G: 0.96±1.44 CF:58.7%	5
Kalantari [34]	2014	Shemiranat, Tehran	T:400 B:204 G:196	dmft:2.46 CF:36.2% Dental caries: 63.4% (in primary dentition)		7
Mehrabkhani [35]	2014	Mashhad m	T:143/ B:80/ G:64	dmft: 6.1±5.4	Dental caries: 81.8% (in primary dentition)	4
Gharibi [36]	2014	Paveh	B:1104	DMFT: 7.91±3.78 DT: 4.71±2.72 MT: 1.70±2.04	FT: 1.50±1.95 CF: 4.4%	9
Mohebbi [37]	2014	Tehran	T: 499/ B:240 G:259	DMFT:11.74±6.78 DT: 4.38±3.67	MT: 4.29±6.41 FT: 3.07±3.23	4
Asgari [38]	2014	Isfahan	T: 592 B:280 G:312	DMFT: T: 2.79±2.7 B: 2.78 G: 2.80	DT: 1.39±1.7 MT: 0.13±0.4 FT: 1.29±2.16 CF: 26% SiC: 5.8±2.1	7
Ahmadi-Motamayel [39]	2013	Hamedan	T:200 B:100 G:100	DMFT: 7.89±3.48 DT: 3.26±2.90	MT: 1.02±1.45 FT: 3.70±3.85	5
Nabipour [40]	2013	Varamin	T:838 B:472 G:366	dmft: T: 3.99±4.036 B: 3.93±4.04 G: 4.07±4.03	Urban: 3.91±4.04 Rural: 4.62±3.98 Dental caries: 78.1% (in primary dentition) CF: 28.2%	7
Nokhostin [41]	2013	Kermanshah	1050	DMFT: T: 0.36±0.8 B: 0.40±0.87 G: 0.31±0.74	dmft: T: 3.04±2.65 B: 3.22±2.71	6
Abedini [42]	2013	Kashan	T:310 B:159 G:151	dmft: T: 1.57±2.3 B: 1.57±2.39	G: 1.58±2.3 CF: 51.3%	6
Faezi [43]	2013	Tehran	G:950	DMFT: 3.56±2.652 in 12 years, 2.961±4.26	in 13 years and 4.38±2.899 in 14 years	7
Hazavei [44]	2012	Hamedan	T:268 B:135 G:133	DMFT: T: 2.25±1.74	B: 1.81±1.65 G: 2.71±1.71	5
Jessri [45]	2012	Tehran	T:1271 B:577 G:694	DMFT:2.70±2.14 dmft: 2.30±2.42	Dental caries: 85.14% (65.9% in primary dentition and 70.2% in permanent dentition)	6
Deyhimi [46]	2011	Isfahan	T:202/ B:82/ G:120	DMFT:4.43±4.494		7
Davari [47]	2011	Yazd	B:475	DMFT: 4.8±3.13 DT: 3.72±2.87 MT: 0.33±0.92 FT: 0.75±1.67 CF:11.5%	DMFT: 4.85±3.51 DT: 3.51±3.1, MT: 0.2±0.7 FT: 1.15±2.1 CF:12.5% Dental caries: 87.5%	4
Eslamipour [48]	2011	Isfahan	T:810 B:371 G:439			7
Ghasempour [49]	2011	Babol	T:600 B:300 G:300	Caries prevalence: T: 65.7% B: 66%	G: 65.4% CF: 34.3%	5
Sadeghi [50]	2011	Rafsanjan, Kerman	T:747 B:353 G:394	DMFT T: 2.83 ± 2.2 B: 3.15 ± 2.1 G: 2.51 ± 2.3	DT: 2.03 ± 2.1 MT: 0.16 ± 0.5 FT: 0.64 ± 1.3 CF: 16.1%	7
Aghighi [51]	2010	Tehran	T:4666 B:2169 G:2497	DMFT: 3.50±2.7 Dental Caries: T: 89.3%	B: 87.4% G: 91.2% CF: 10.7%	5
Karimi Zarchi [52]	2010	Tehran	T:401 B:202 G:199	DMFT: T:1.01±1.47 B: 0.93±1.44 G: 1.08±1.49 DT:1.01±1.47 MT:0.61±1.51 FT:0.42±0.95	dmft: T: 5.29±3.52 B: 5.48±3.61 G: 5.09±0.42 dt: 3.01±2.95 mt: 0.47±0.88 ft: 1.78±2.0	7
Mohebbi [53]	2009	Gonabad, Khorasan Razavi	T:529 B:266 G:263	DMFT: T:1.04±0.22 B:1.02±0.17 G:1.07±0.28 DT: 0.78±0.38 MT:0.17±0.18 FT:0.09±0.12	dmft: T: 3.86±1.11 B: 3.74±1.07 G: 3.99±1.16 dt: 2.62±1.65 mt: 0.77±0.96 ft: 0.47±0.84 CF: 8.3%	8
Torabi [54]	2009	Kerman	T:154/ M:60/ F:94	DMFT:10.88±6.47 Men:11.05±6.19	Femal:10.85±6.65 CF: 0%	6
Sadeghi [55]	2009	Rafsanjan, Kerman	T:353 B:180 G:173	DMFT: T: 2.46 ± 2 B: 2.78 ± 1.8	G: 2.13 ± 2.1 CF: 20.7%	5
Hematyar [56]	2009	Tehran	T:200/B:106/G:94	dmft: 2.32±2.56	Dental Caries: 63.5 %	7
Seyed Akhavan [57]	2008	Karaj	T:768 B:384 G:384	DMFT: B: 3.59±2.59 G: 2.67±2.21	CF: B: 13.5% G: 21.9%	7
Hamissi [58]	2008	Qazvin	T:780 B:390 G:390	DMFT: 2.71±0.86 B: 2.88±0.61 G: 2.54±0.71	DT: 2.23±0.90 MT: 0.23±0.86 FT: 0.25±0.07 CF: 24.5%	5
Yazdani [59]	2008	Tehran	T:500 B:260 G:246	DMFT; T: 2.1 B:2.0, G:2.2 DT=0.9, MT= 0.2, and	FT=1.0 SiC:5.2 CF; T: 40%, B:44% and G:37%	7
Eskandarian [60]	2006	Shiraz	T:280/ B:150/ G:130	dmft: 2.87±3.26		6
Broumand [61]	2006	Tehran	T:170/B:88/G:82	DMFT:3.117	Dental Caries: 70.8%/CF: 29.2%	4
Meyer-Lueckel [62]	2006	Tehran, Semnan	T:523 B:256 G:267	1) in 6-year-old pupils in Tehran dmft: T: 3.3±2.7 B: 3.0±2.1 G: 3.5±3.1 dt:1.3, mt: 0.1 and ft:1.7, CF: 17% 2) in 6-year-old pupils in Semnan dmft: T: 3.3±2.9 B: 4.2±3.1 G: 2.4±2.5 dt:3.0, mt:0.2 and ft: 0.1, CF: 19% CF: 30%	3) in 9-year-old pupils in Tehran, dmft: T: 2.6±2.2; B: 3.0±2.5; G: 2.4±2.1 dt:1.0, mt:NR and ft:1.4, CF:23% 4) in 9-year-old pupils in Semnan, dmft: T: 2.1±2 B: 2.5±2.0 G: 2.1±2.0 dt: 2.1, mt: NR and ft: 0.2,	4
Javadinejad [63]	2006	Isfahan	340	DMFT: 2.4	SiC:4.6	4
Salem [64]	2006	Langrood, Lahijan, Siahkal	T:885 B:454 G:431	1) Langrood DMFT; T:1.65±1.61 B:1.68±1.61 G:1.61±1.5 DT:1.39, MT:0.06 and FT:0.2 CF: 33.3%	2) Lahijan /DMFT; T:1.49±1.56; B:1.29±1.44; G:1.70±1.68; DT:1.1, MT:0.02 and FT:0.37; CF: 39.3% 3) Siahkal DMFT; T:1.60±1.4; B:1.45±1.38 G:1.77±1.51; DT:1.38, MT:0.15 and FT:0.07; CF: 34.4%	6
Houshmand [65]	2006	Hamedan	T:238/ B:113/ G:125	Caries prevalence: T:85.3% B:85.8%	G:84.8% CF: 14.7%	4
Ajami [66]	2006	Mashhad	T:1938 B:948 G:990	T:1938 B:948 G:990	1)Teheran DMFT:1.10 ±1.50 DFS:1.59 ±2.59 CF: 52.3% 2)Villages near Esfahan DMFT:0.38 ±0.82 DFS: 0.70 ±1.73 CF: 77.3%	4
Momeni [67]	2006	Teheran Isfahan	1102			4
Toomarian [68]	2005	Qom	T:300 B:150 G:150	DMFT; T:2.28 ±2.1 B:2.78 ±2.18	G:2.87 ±2.03 SiC:5.15±1.45 CF: 10.7%	7
Vejdani [69]	2005	Guilan	T:261/B:111/G:147	dmft:1.79	CF: 36.4% ECC: 28%	5
Daneshkazemi [70]	2005	Yazd	T:1223 B:654 G:569	DMFT: T:1.8±1.75 B:1.95±1.91	G:1.65±1.55 Dental caries: 74.73% CF; T: 28.6%, B: 27.25%, G: 30.05%	5
Kazerouni [71]	2005	Tehran	T:1024 B:528 G:496	DMFT:1.5±1.5 DT:1.9±1.2 MT:0.1±0.5 FT:0.2±0.7	dmft:6.2±2.7 dt:3.6±2.6 mt:1.2±1.6 ft:1.2±1.9 CF: 22.1%	6
Davari [72]	2004	Ardakan	T:607 B:303 G:304	DMFT:1.45±1.5 DT:1.141±1.41 MT:0.0779±0.34	FT:0.235±0.67 CF: 41%	6
Ramezani [73]	2004	Dayer, Boshehr	T:506 B:281 G:225	DMFT; T:1.8±1.7, B:1.98±1.67 G:1.65±1.76	DT=1.62±1.66, MT= 0.04±0.22, FT=0.13±0.45	4
Ghandehari [74]	2003	Tehran	T:400 B:240 G:160	Dmft T: 2.45 B:2.41 G:2.51	dt: 1.95, mt:0.43, ft:0.07 CF: 48.3%	5
Ghandehari-Motlagh [75]	2003	Guilan	T:144 B:76 G:68	DMFT T:1.68±1.78 B:1.44±1.64 G:1.96±1.91	DT:1.47±1.65 MT:0.28±0.14 FT:0.18±0.67 CF: 36.1%	6
Saneie [76]	2003	Khomain	T:713 B:365 G:348	DMFT T: 0.15±0.53 B:0.12±0.47 G:0.18±0.59 Urban: 0.15±0.53 Rural: 0.19±0.60	dental caries: 84.1% in primary dentition and11.8% in permanent dentition /dmft T:4.02±3.34 B: 4.26±3.57 G: 3.76±3.08 Urban: 3.88±3.38 Rural: 4.30±3.27	8
Fani [77]	2003	Bavanat, Fars	T:407/B:271/ G:136	DMFT/ T:2.25±1.9 B:2.33±1.98	G:2.09±1.73 CF: 25.6%	8
Mortazavi [78]	2002	Boshehr	T:506/ B:281/ G:225	DMFT:1.8±2.33 DT: 1.62±1.66	MT: 0.04±0.22 FT: 0.13±0.45	3
Memar [79]	2000	Sanandaj	T:439/ B:224/ G:215	DMFT T:2.6±2.33 B: 2.67±1.79	G: 2.52±1.84 CF: 15.7%	8
Eskandarizadeh [80]	1999	Sirjan	T:300 B:15 G:150	Caries prevalence: 65.3%	CF ;T: 34.7% B:39.3%, G:30%	4
Gholami [81]	1998	Tehran	254	DMFT:5.5±3.6	CF: 57%	3

Abbreviations: NOS: Newcastle–Ottawa Scale; T: total; B: boy: G: girl; DMFT: the decayed, missing, filled teeth for permanent teeth; DT: decayed teeth in permanent teeth; MT: missing teeth in permanent teeth; FT: filled teeth in permanent teeth; dmft: the decayed, missing, filled teeth for primary teeth; dt: decayed teeth in primary teeth; mt: missing teeth in primary teeth; ft: filled teeth in primary teeth; CF: caries free; SiC: Significant Caries; ECC: Early Childhood Caries; MMS: Multistage Stratified Sampling; SCS: Stratified Cluster sampling.

Most studies were of strong or moderate quality and only three studies deemed to be rated weak. Almost all studies were conducted in children and adolescents (age range of fewer than 18 years old) except 4 studies that were conducted on adult population. The sample size of those studies in adult population was 1,055 individuals (482 male and 573 female). While, sample size for children and adolescents population was 45,493 individuals (49.61% boys); in overall, considering all the included studies, the total number of participants was 46,548 individuals. 

Given the limited studies in adult age groups, the data were not sufficient to compute many variables and factors; so, only the mean DMFT was calculated in adult age groups and other results and analyzes were only calculated in the age group less than 18 years old.

### Main analysis

**Figure 2:**
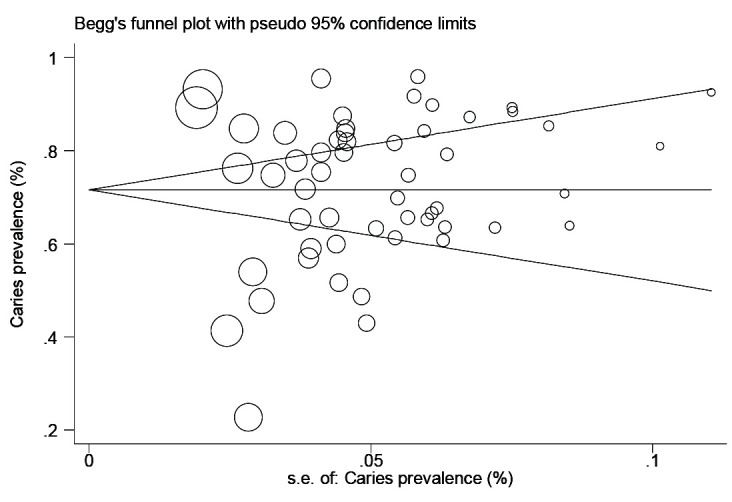
The mean of dmft index in Iran based on random effects model. The midpoint of each segment is the estimate of prevalence and segment lengths show the 95% CI for each study. The diamond mark shows the prevalence in the country for all studies.

Table 2 presents the pooled estimations of the prevalence of dental caries status and DMFT/dmft data using meta-analysis of data extracted from studies that met the fifty-four studies reporting dental caries (deciduous and permanent teeth) were collected for calculating the overall estimation of the dental caries prevalence in Iran. The results show the dental caries rate (deciduous and permanent teeth) in population younger than 18 years old for all studies in all regions of Iran based on random effect model to be 72.8% with 95% confidence interval (69.2-76.4%). High heterogeneity was found as demonstrated by Q-value of 5588.13 (df= 53) and I^2^ of 99.1% (*p*<0.00). We found the prevalence of dental caries for boys and girls was 75.3% and 74.9% respectively, almost similar in both sexes. In addition, the proportion of decayed (d), missing (m), and filled (f) teeth in primary teeth were 70%, 0.9% and 14%, respectively. Frequency of decayed (D), missing (M) and filled (F) teeth in permanent teeth were 59%, 6% and 21%, respectively. The total prevalence of caries-free (CF) from meta-analysis of data extracted from the reviewed studies was 27.2% (95%CI=23.5-30.8%) and this rate in boys and girls was 24.33% and 26%, respectively. The results of meta-analysis showed that the mean of dental caries was 2.33(95% CI, 2.12–2.54) based on DMFT values and 3.86 (95% CI, 3.49–4.22) based on the dmft values (Figure 2). 

Visual inspection of these forest plots indicates a high level of heterogeneity; I^2^ values for DMFT and dmft were 99.7%, and 99.5%, respectively. In addition, chi-square test provided a significant p Value (<0.001), which confirms the heterogeneity between studies. Data on the mean DMFT in adult were available for four studies and we were able to calculate mean DMFT; accordingly mean DMFT index based on a random effect model was 8.75 (95% CI, 5.47–12.03), which indicates that heterogeneity was significant (I^2^ = 99%, *p*< 0.001).

We found that the mean of DMFT indexes was similar based on sex, and differed based on urban/rural residence. The mean DMFT was higher in rural than urban regions (2.35 vs. 1.89). Similar results were obtained for dmft indexes (Table 2). The average of decayed (d), missing (m), and filled (f) teeth in primary and in permanent teeth are shown in Table 2. These rates were almost similar in boys and girls, expect for filled teeth in primary (ft.) and permanent teeth (FT). We observed that the mean both ft. and FT were significantly higher in girls than boys (0.50 vs. 0.36 for dmft and 0.38 vs. 0.23 for DMFT). Eight studies reporting significant caries (Sic) were collected for calculating the overall estimation of this index; our results showed that the mean Sic was calculated as 6.88 (95%CI=5.34-8.52). 

**Table 2:** Dental caries status and DMFT/dmft data using Random Effect Meta-Analysis.

Dental caries index	Number of studies	Prevalence %	Confidence interval 95% (CI%95)	Heterogeneity index I^2^ (%)
Dental caries				
Total	54	72.8	69.2- 76.4	99.1%
Boys	22	75.3	70.6- 80	97.55%
Girls	22	74.9	70.3 – 79.5	97.7%
DT	13	59.0	44.0 – 73.0	99.7%
MT	10	6.0	4.0 – 8.0	92.3%
FT	10	21.0	12.0 – 30.0	99.1%
dt	7	70.0	53.0 – 87.0	99.6%
mt	6	0.9	4.0 – 13.0	96.4%
ft	7	14.0	9.0 – 18.0	89.4%
CF				
Total	54	27.2	23.5-30.8	99.1%
Boys	23	24.33	19.8-28.9	97.5%
Gils	23	26.0	21.0-30.0	97.8%
The mean dmft\DMFT				
	Number of studies	Mean	(95% CI)	I^2^ (%)
DMFT				
Total	48	2.33	2.12 – 2.54	99.7%
Boys	29	1.97	1.75 – 2.19	99.7%
Gils	29	1.94	1.71 – 2.16	99.5%
Urban	5	1.89	1.21 – 2.57	99.8%
Rural	5	2.35	1.46 – 3.24	99.8%
DT				
Total	22	1.95	1.59 – 2.31	99.7%
Boys	8	1.40	0.91 – 1.88	99.7%
Gils	8	1.48	0.96 – 2.0	95.5%
MT				
Total	18	0.20	0.15 – 0.25	99.55%
Boys	8	0.13	0.03 – 0.23	99.7%
Gils	8	0.11	0.03 – 0.18	99.8%
FT				
Total	17	0.32	0.26 – 0.38	99.4%
Boys	8	0.23	0.17 – 30.0	97.4%
Gils	8	0.38	0.26 – 0.50	99%
dmft				
Total	30	3.86	3.49 – 4.22	99.5%
Boys	19	3.72	3.35 – 4.09	98.7%
Gils	19	3.54	2.86 – 4.23	99.8%
Urban	4	4.25	3.88 – 4.62	89.4%
Rural	4	4.98	4.45 – 5.51	95.24%
dt				
Total	11	3.49	2.87 – 4.11	99.3%
Boys	8	2.54	1.79 – 3.30	98.9%
Gils	8	2.50	2.09 – 2.91	99.9%
mt				
Total	9	0.50	0.30 – 0.69	98.7%
Boys	8	0.39	0.22 – 0.57	99.6%
Gils	8	0.33	0.17 – 0.49	99.6%
ft				
Total	9	0.67	0.50 – 0.84	99.0%
Boys	8	0.36	0.25 – 0.47	96.9%
Gils	8	0.50	0.32 – 0.68	98.5%
SiC				
Total	8	6.88	5.34 – 8.52	99.9%
Boys	3	7.65	5.20 – 10.10	99.7%
Gils	3	7.48	4.86 – 10.10	99.8%

### The factors affecting on dental caries

We performed a separate meta-analysis for the association between the children’s demographic factors and their dental caries experience (DMFT/dmft>0). The results are presented in Table 3; as shown, the mean dental caries was higher in those children whose family had low socioeconomic position and income than those their family had high socioeconomic position (3.76 vs. 2.93) and higher income (2.39 vs. 2.31). Moreover, lower scores of caries index were found in the children who had academic educated (2.26 vs. 3.33) and employed parents (2.38 vs. 3.05). The average dental caries in children from crowded families was greater than sparsely populated families (2.88 vs. 2.0). We found that excessive carbohydrate intake is significantly increase the incidence of dental caries; so that, dental caries mean was 4.33 in children who had high levels of carbohydrate intake and 2.04 in those who were less likely to use it. In addition, the frequency of children’s tooth brushing was related to dental caries experience; children with more tooth brushing times showed less caries (1.94 vs. 2.99). Average of dental caries in children who had more dental visits was lower than those whom had not visited a dentist (2.59 vs. 2.66).

### Sensitivity and Subgroup Analyses

Due to high heterogeneity among reviewed studies, we conducted subgroup analyses based on age group and region to minimize heterogeneity. Table 4 presents the results of subgroup analyses. Studies based on age group were divided to five groups as follow: younger than 6, 6, 9, 12 and 12-18 years; and the dental caries prevalence was 71%, 56%, 73%, 68% and 72%, respectively. The prevalence was almost similar in all other age groups expect for the group of 6-year-old subjects who had the smallest frequency of dental caries (56%). The mean DMFT index was 0.85, 2.13, 2.52, and 3.71 in age group6, 9, 12 and 12-18 years, respectively. We found a direct relationship between DMFT index and age, as the age increased, the mean of DMFT increased. We also observed an inverse relationship between dmft and age; the average of dmft index was 3.99, 4.92, 3.80 and 0.76 in age group ˂ 6, 6, 9, and 12 years, respectively. Mean Sic was higher in age group 12-18 years than age group 12 years (7.71 vs. 6.73) which indicates that it increases with age. In this study, we also performed a subgroup analyses according to region of residence and found that the average of dental caries, DMFT and dmft values were differ based on region of residence (data not shown). A sensitive analysis was also performed in the current research to find the effect of influential studies on the overall results. We found no special study to change the overall results.

**Table 3:** The factors affecting on dental caries in population age range of fewer than 18 years old in Iran.

Factors	Number of studies	The mean DMFT/dmft	I^2^ (%)
High socioeconomic position	3	2.93 (0.85 – 5.01)	99.7%
Low socioeconomic position	3	3.67 (0.34 – 7.0)	99.8%
Education, Low	15	3.33 (2.67 – 4.00)	99.3%
Education, Academic	15	2.26 (1.72 – 2.80)	99.4%
Income, Salary	2	2.31 (1.81 – 2.82)	99.55
Income, Low	2	2.39 (1.51 – 3.28)	97.8%
Occupation, Unemployed	5	3.05 (1.69 – 4.41)	99.6%
Occupation, Employed	5	2.38 (1.50 – 3.27)	99.6%
Children in family-Maximum	9	2.88 (2.16 – 3.59)	98.9%
Children in family- Minimum	9	2.00 (1.49 – 2.51)	98.9%
Carbohydrate consumption, Maximum	2	4.33 (3.02 – 5.65)	98.8%
Carbohydrate consumption, Minimum	2	2.04 (-0.82, 4.90)	99.8%
Toothbrush, Maximum	13	1.94 (1.34 – 2.53)	99.3%
Toothbrush, Minimum	13	2.99 (2.05 – 3.94)	99.7%
Dental visits- Maximum	5	2.59 (1.87 – 3.32)	98.1%
Dental visits- Minimum	5	2.66 (2.15 – 3.16)	96.5%

**Table 4:** Distribution of Dental caries and DMFT/dmft data according to age.

Variables	˂6 years	6 years	9 years	12 years	12-18 years
Dental caries (%)	71 (65-77)	56 (36-75)	73 (54-91)	68 (61-76)	72 (62-82)
DMFT(mean)	-	0.85(0.33-1.37)	2.13(1.88-2.39)	2.52(2.17-2.86)	3.71(3.19- 4.23)
dmft (mean)	3.99(3.33-4.65)	4.92(4.30-5.53)	3.80(3.37-4.24)	0.76(0.42-1.10)	-
SiC (mean)	-	-	-	6.73(5.37-8.10)	7.71(6.61- 8.9)

### Publication Bias

**Figure 3:**
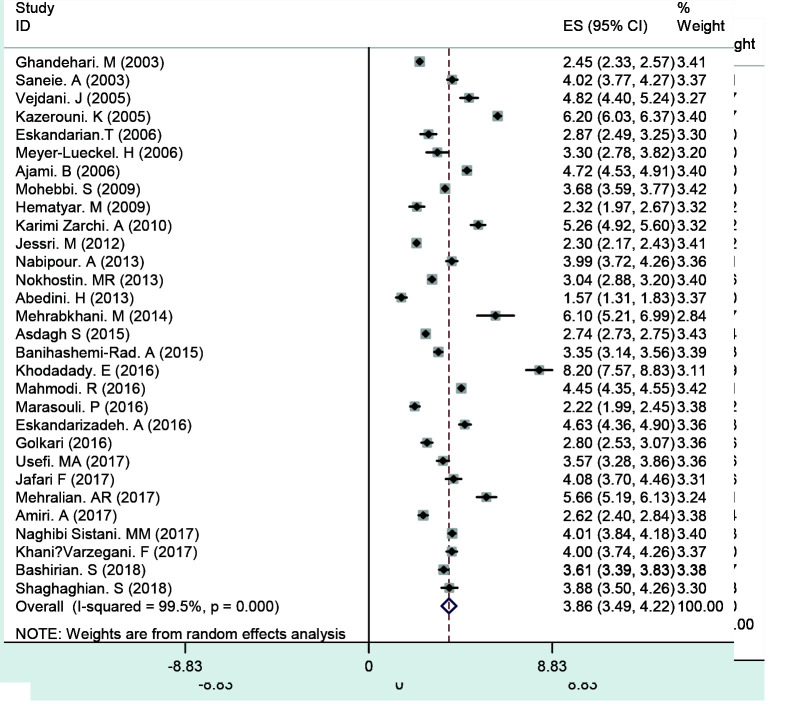
Funnel plot for checking publication bias

Publication bias was checked using a Begg’s funnel plot (Figure 3). An asymmetric funnel plot indicates no publication or study bias; so, the effect of bias was not significant when Begg’s funnel plot evaluated (p= 0.633). Egger’s test was also used for confirmation of the absence of publication bias and result showed no evidence of publication bias in this study (t= 0.11, p= 0.913) (Figure 3).

## Discussion

In the present study, we considered the dental caries status and its confounding factors in our systematic search.

The total rate of dental caries was 72.8% (d: 70%, m: 0.9%, and f: 14%, D: 59%, M: 6%, and F: 21%, respectively). The mean of DMFT values was 2.33 (D: 1.95, M: 0.20, and F: 0.32, respectively) and dmft values was 3.86 (d: 3.49, m: 0.50, and f: 0.67, respectively). These findings showed that the prevalence of dental caries among children and adolescents (< 18 years of age] in Iran was high. To the best of our knowledge, this study is the first research that have provided information using meta-analysis regarding the dental caries status, and associated factors in Iran. Already, several nationwide surveys have been carried out to describe the oral health status of children in the Islamic Republic of Iran; in the first national oral health survey in 1998, the percentage of dental caries among 6- and 9-year-old children in 1998–99 was 86.2% and 88.5% respectively. The mean dmft were 1.8, 4.8, and 0.9 for 6, 9, and 12 year-olds. In addition, the mean DMFT were 0.2, 3.4, and 1.5 for 6, 9, and 12-year-olds respectively [ 82 ]. A second nationwide survey in 2004, using WHO guidelines, were reported the dental caries rates (52%, 89%, 90% and 68%) and the mean dmft value (1.9, 5.0, 3.6 and 0.6 P) in 3, 6, 9 and 12 years old children; also the mean DMFT indices were 0.2, 0.9 and 1.9 for 6, 9 and 12-year-old children, respectively [ 83 ]. Caries rates in another descriptive nationwide study in 2009‒2010 among first-year students of elementary, junior and high schools in 32 provinces in Iran were 59.06%, 47.23% and 40.98% respectively [ 84 ].These data supported by the findings of the present study and showed that the dental caries status in Iran was expected to be bad. Similar to our findings, the caries prevalence in the reported studies of Asian countries was high and it is of special concern for Middle Eastern populations. The caries prevalence and mean dmft score among preschool children in Southeast Asia were 79% and 5.1, respectively [ 85 , 87 ]. A systematic review in Arab League countries reported a mean dmft score of 4.5 in 2-7 years old children [ 86 ]. Another systematic review and meta-analysis of 34 reviewed studies found the overall mean dmft in the primary teeth was 5.14 with prevalence of 80.9% in pre-school children in the Gulf Cooperation Council area [ 87 ]. In addition, the prevalence of dental caries in Iran is much closer to that in some Latin American countries. A systematic review and meta-analysis in Latin American and Caribbean children from seventy-five studies showed that caries prevalence in Brazil and other investigated countries for primary teeth in 5-6 years old children were 52% and 70%, and for permanent teeth in 11-12 years old children were 56% and 63%, respectively [ 88 ]. Also the prevalence of early childhood caries among 5-6 year-old children in South Africa and Swaziland, was 57%, and the mean dmft score was 3.1 [ 85 ].These data showed the inappropriate dental caries status in Iran and other developing countries. Unlikely, the prevalence of dental caries in developed countries was much lower; the caries prevalence in schoolchildren was 26.2% in Germany [ 89 ], 30% in France [ 90 ], 27.6% in Great Britain [ 91 ], 23% in USA [ 92 ] and 28% in UK [ 93 ]. Comparing these findings with our data showed that the prevalence rate of dental caries among children is markedly higher in Iran compared to developed countries.

The high prevalence and experience of dental caries in Iran compared to developed countries is because of ineffective oral health national preventive programs and lack of educational measures [ 94 , 95 ], which implemented in developed countries. One of the other reasons might be explained by increased fluoride consumption [ 96 ]. Current evidence shows that adequate fluoride in drinking water can help prevent dental carries [ 97 ].This is while fluoride concentration in drinking water in most regions in Iran is lower than the standard level [ 98 ]. In addition, the results of our meta-analysis showed that frequency of tooth brushing is related to dental caries experience. Thus, poor oral health and low oral hygiene could to some extend explain the reason of observed high prevalence of dental caries in Iran. Furthermore, we observed that children had a large proportion of untreated dental caries and a low proportion of filled teeth (data are shown in table 3), this finding indicate insufficient attention of parents to preventive and restorative needs of their children. These results explain the inappropriate dental caries status in Iran, which is consistent with studies, conducted in developing and under, developed countries, and contradict with those reported in developed countries. 

We observed no differences in caries status between boys and girls; this figure was observed regarding all components' dmft and DMFT expects for ft. and FT; our results showed a high proportion of the mean both ft. and FT in girls than boys (table 3). The increase in the number of filled teeth in girls than boys may influence by a variety of behavioral, environmental, and physiological factors [ 99 ]; this difference can be also due to sex discrimination because in our country parents commonly pay more attention to the appearance of their girls than boys do [ 13 , 14 ].

We also observed the chance of developing DMFT increases by age and this trend was found in most cohorts over time [ 100 ]. Caries experience is more likely to increase with increasing age of children because as the age increases, teeth exposure to cariogenic diet increases, which accelerates the decay and erosion of teeth [ 101 , 102 ]. Our study, similar to others showed that the mean dmft was more prevalent in younger age groups because of lack of knowledge on efficient preventive behaviors in younger age, inappropriate eating habits such as frequent consumption of sugary foods and snacks, and higher caries resistance in permanent teeth compared to primary teeth [ 103 , 104 ].

### Relationship between the children’s demographic factors and oral hygiene with their dental caries status

We observed an invert association between parent’s education and their children's dental caries. This finding is accordance to previous studies [ 105 , 106 ]. The lack of awareness and necessary skills of parents about the oral hygiene can be an explanation for this finding. Educational background also affects oral health literacy, dietary habits, tooth cleaning patterns, and health service utilization [ 105 , 106 ]. The results of our study also demonstrated better dental health status in the children whom their parent had a governmental occupation and higher income than others. A possible explanation is that having a governmental occupations frequently increase income, thus families with higher income are able to spend more budgets for access to preventive means such as toothpastes, dental floss, and health service utilization [ 107 ]. We observed caries experience was more frequent among children from a poorer socioeconomic position. A systematic review and meta-analysis study showed that low socioeconomic position is significantly associated with a higher risk of having caries lesions or experience [ 108 ]. In most developed countries, dental services are universally available, while they so expensive in developing countries like Iran and access to them might be so difficult for people from a lower socioeconomic background [ 109 ]. Our meta-analysis indicated that frequency of children’s tooth brushing is related to dental caries experience. A systematic review and meta-analysis study showed that infrequent brushers were at greater risk for carious lesions incidence compared to those who were brushing frequently; tooth brushing removes dental biofilm and in this way reduces the incidence and development of carious lesions [ 110 ]. The highly observed prevalence of dental caries in this study demonstrates infrequent and non-acceptable effectiveness of tooth brushing in children. This highlights inadequate educational program about tooth brushing skill and insufficient attention of the parents to children’s oral hygiene. Similar to other studies, we observed that children living in the rural residence had a worse dental status compared to those children living in urban regions. This could be explained by difference in the context of cultural, nutritional habits, socioeconomic status, geographical factors, and so forth; rural children generally have low socioeconomic position and family income, low educated and unemployed parents, crowded families, and difficulties in access to dental services [ 111 , 112 ]. 

### Strengths and Limitations

This is the first meta-analysis of dental caries status and associated factors in Iran, which have provided valuable information in a very large sample size. As dental caries is one of the most expensive diseases to treat, the results of this study have major implications for oral health policy and planning health services. This study had also some limitations; first, we sought to identify all the published studies for dental caries, however it is possible some papers have been missed due to time and resource constraints. Secondly, the quality assessment of selected studies for this study may be subject to criticism because there is possible measurement error in assessing quality of the studies. Third, dmft/DMFT index is used in this meta-analysis; these indices underestimate the value of dental caries than the actual value because it is inefficient in detecting dental cavities, and unable in determining the non-cavitated lesions [ 113 ]. In some countries, other indices such as International Caries Detection and Assessment System (ICDAS) were used for assessing dental caries but this approach is not widely used in Iran. Thus, we did not obtain adequate data for dental caries analysis based on ICDAS, although the DMFT/dmft index is recommended for assessing dental caries by WHO and is still considered as a valid approach in many countries [ 114 , 115 ]. Other limitations in most meta-analysis studies are heterogeneity. We conducted subgroup meta-analyses and sensitivity analyses to explore this heterogeneity. However, in most cases, heterogeneity could not be explained, accordingly, a random effects model was applied to incorporate heterogeneity into our analyses. Furthermore, the possible effects of publication bias inherent in any meta-analysis cannot be ruled out.

## Conclusion

The present study showed high prevalence of dental caries among children and adolescents (<18 years of age] in Iran. Moreover, we found the percentage of children with experience of dental caries was too high. These data show the inappropriate dental caries status in Iran compared to developed countries and illustrate ineffective oral health national preventive programs, lack of educational measures, inadequate fluoride in drinking water, poor oral health, low oral hygiene, and insufficient attention of parents to needs of their children for preventive and restorative dental treatments. Our findings indicate that educational programs about oral health in Iran is not adequate and new preventive procedures, interventional measures, practical educational programs, and modern therapeutic methods are needed to improve oral health status specially for the children whose family have low socioeconomic position and income.
